# NAD1 Controls Defense-Like Responses in *Medicago truncatula* Symbiotic Nitrogen Fixing Nodules Following Rhizobial Colonization in a BacA-Independent Manner

**DOI:** 10.3390/genes8120387

**Published:** 2017-12-14

**Authors:** Ágota Domonkos, Szilárd Kovács, Anikó Gombár, Ernő Kiss, Beatrix Horváth, Gyöngyi Z. Kováts, Attila Farkas, Mónika T. Tóth, Ferhan Ayaydin, Károly Bóka, Lili Fodor, Pascal Ratet, Attila Kereszt, Gabriella Endre, Péter Kaló

**Affiliations:** 1 National Agricultural and Innovation Center, Agricultural Biotechnology Institute, 2100 Gödöllő, Hungary; domonkos.agota@abc.naik.hu (A.D.); gombar.aniko@abc.naik.hu (A.G.); horvath.beatrix@abc.naik.hu (B.H.); kovats.gyongyi@abc.naik.hu (G.Z.K.); toth.monika.tunde@abc.naik.hu (M.T.T.); fodor.lili@abc.naik.hu (L.F.); kalo.peter@abc.naik.hu (P.K.); 2 Institute of Plant Biology, Biological Research Center, 6726 Szeged, Hungary; kovacs.szilard@brc.mta.hu (S.K.); farkasa8@gmail.com (A.F.); kereszta@gmail.com (A.K.); endre.gabriella@brc.mta.hu (G.E.); 3 Institute of Genetics, Biological Research Center, 6726 Szeged, Hungary; kiss.erno@brc.mta.hu; 4 Cellular Imaging Laboratory, Biological Research Center, 6726 Szeged, Hungary; aferhan@gmail.com; 5 Department of Plant Anatomy, Eötvös Loránd University, 1117 Budapest, Hungary; karolyboka@caesar.elte.hu; 6 Institute of Plant Sciences Paris-Saclay IPS2, CNRS, INRA, Université Paris-Sud, Université Evry, Université Paris-Saclay, Bâtiment 630, 91405 Orsay, France; pascal.ratet@ips2.universite-paris-saclay.fr; 7 Institute of Plant Sciences Paris-Saclay IPS2, Paris Diderot, Sorbonne Paris-Cité, Bâtiment 630, 91405 Orsay, France

**Keywords:** defense response, nodule, symbiosis, nitrogen fixation, legume, *Medicago truncatula*, rhizobia

## Abstract

Legumes form endosymbiotic interaction with host compatible rhizobia, resulting in the development of nitrogen-fixing root nodules. Within symbiotic nodules, rhizobia are intracellularly accommodated in plant-derived membrane compartments, termed symbiosomes. In mature nodule, the massively colonized cells tolerate the existence of rhizobia without manifestation of visible defense responses, indicating the suppression of plant immunity in the nodule in the favur of the symbiotic partner. *Medicago truncatula* *DNF2* (defective in nitrogen fixation 2) and *NAD1* (nodules with activated defense 1) genes are essential for the control of plant defense during the colonization of the nitrogen-fixing nodule and are required for bacteroid persistence. The previously identified nodule-specific *NAD1* gene encodes a protein of unknown function. Herein, we present the analysis of novel *NAD1* mutant alleles to better understand the function of NAD1 in the repression of immune responses in symbiotic nodules. By exploiting the advantage of plant double and rhizobial mutants defective in establishing nitrogen-fixing symbiotic interaction, we show that NAD1 functions following the release of rhizobia from the infection threads and colonization of nodule cells. The suppression of plant defense is self-dependent of the differentiation status of the rhizobia. The corresponding phenotype of *nad1* and *dnf2* mutants and the similarity in the induction of defense-associated genes in both mutants suggest that NAD1 and DNF2 operate close together in the same pathway controlling defense responses in symbiotic nodules.

## 1. Introduction

*Medicago truncatula* and other leguminous plants are able to establish nitrogen-fixing symbiotic associations with soil bacteria that are belonging to the genus rhizobia. The symbiotic interaction between the rhizobia and the host plant is initiated by exchange of chemical signals between the two partners resulting in the formation the root nodules, wherein nitrogen fixation takes place [1,2]. Plant flavonoids secreted into the rhizosphere induce the production of the bacterial signal molecule, the nodulation factor (NF) that triggers morphological changes, root hair curling and cortical cell division, and physiological responses, such as ion fluxes, calcium oscillations, and transcriptional activation of symbiosis associated genes [1], in the host plant. Rhizobia are required for the initiation and growth of infection threads (ITs) that develop towards the newly formed nodule primordia by penetrating the outer cortical cell layers [3]. 

When ITs reach nodule primordia, bacteria are released from these ITs and colonize the host cells through endocytosis. The bacteria are accommodated in a cytoplasmic structure referred to as the symbiosome that is delimited by a plant derived peribacteroid membrane. 

The symbiotic interaction between *Sinorhizobium* (*Ensifer*) sp. and *M. truncatula* leads to the formation of cylindrical-shaped indeterminate nodules that possess a persistent meristem. The mature indeterminate nodules are composed of histological zones containing nodule cells and rhizobia at different developmental states [4]. The continuously active meristem (zone I, ZI) produces cells, which internalize rhizobia in the infection zone (ZII), both colonized host cells, and rhizobia differentiate in interzone (IZ), differentiated bacteroids reduce nitrogen to ammonium in zone III, and plant cell content and bacteroids are being degraded in the senescence zone (ZIV) proximal to the root tissue. In indeterminate nodules, bacteria undergo irreversible morphological changes (shape alteration, enlargement, and polyploidization) [5]. This terminal differentiation is governed by nodule-specific cysteine-rich antimicrobial peptides (NCRs), which are produced in infected host cells [6], and are targeted to symbiosomes. This process requires the *M. truncatula* DNF1 protein, a nodule-specific subunit of a signal peptidase [7]. The bacterial BacA protein is required for *Sinorhizobium meliloti* differentiation and chronic infection of *M. truncatula* nodules. *S. meliloti* strain deficient in BacA is susceptible for the antimicrobial effect of NCRs and rhizobia are rapidly killed once they are released from ITs [8].

During the infection process and internalization of rhizobia in the host cells, bacteria invade plant tissues without provoking chronic defense responses, indicating the control of plant immunity in nodules during the symbiotic process. For instance, transcriptome studies revealed the induction of defense-associated genes in legumes few hours post inoculation with compatible rhizobia, and this activation was attenuated hours later [9,10,11]. The generation of reactive oxygen species (ROS) is an important component of plant defense [12]. The abortion of infection threads, the outcome of the autoinhibition process to control the number of infection events is coupled with presence of polyphenolics, and is accompanied with hypersensitive reaction, showing a role for plant defense responses during infection [13]. The control of hydrogen peroxide level is required for the fine tuning of the infection process [14], but ROS act as signaling molecules during the rhizobial invasion and moreover regulate nodule senescence [15]. *S. meliloti exoY* mutant, defective in succinoglycan (exopolysaccharide I; EPS-I) production, induces the formation of root hair curling, but is deficient in infection thread formation [16]. *M. truncatula* plants that are inoculated with the succinoglycan-deficient *S. meliloti* show the induction of plant defense responses [17] and strong activation of defense-related genes in *M. truncatula* roots [16], suggesting a function for EPS in suppression of defense-responses during the infection process. 

The suppression of plant immunity is required not only during the early infection events but later as well since nodule cells have to tolerate the massive colonization of rhizobia during the establishment and maintenance of the nitrogen-fixing rhizobium–legume symbiosis [18]. *M. truncatula* mutants that are deficient in the *DNF2* (*Defective in Nitrogen Fixation 2*), *SymCRK* (*Symbiotic Cysteine-rich Receptor-like Kinase*), *NAD1* (*Nodules with Activated Defense 1*) and *RSD* (*Regulator of Symbiosome Differentiation*) genes display strong defense-related responses, indicating the loss of control over suppression of plant immunity [19,20,21,22]. *DNF2* and *SymCRK* encode a phospholipase-like protein and cysteine-rich receptor-like kinase with a non-arginine–aspartate motif, respectively, whereas, RSD encodes a C_2_H_2_ transcription factor. Their functions have not been clearly determined in repressing plant defense response, but a recent model has proposed the successive operation of DNF2, SymCRK, and RSD to regulate rhizobial colonization and persistence in *M. truncatula* nodules [23].

Finally, the *nad1* mutant showed severe necrotic phenotype with low bacterial occupancy of the nodules, indicating the requirement of NAD1 in the maintenance of nitrogen fixing endosymbiosis [21]. The nodule specific *M. truncatula NAD1* encodes a protein with unknown function and its homologs are specifically present in plants establishing root nodule symbiosis.

Here, we report the phenotypic analysis and the functional study of additional mutant alleles of *NAD1*. To better understand the function of NAD1 in suppression of plant immunity during nodule differentiation, the transcriptional analysis of defense-associated genes and the accumulation of phenolic compounds were monitored in single and double *M. truncatula* symbiotic mutants, defective in establishing effective nitrogen-fixing interaction, inoculated with wild-type and symbiotic mutant rhizobia.

## 2. Materials and Methods

### 2.1. Plant Material, Growth Condition and Bacterial Strains

*Medicago truncatula* Jemalong, A20 and 2HA genotypes were used as wild-type controls in phenotypic characterization and genetic mapping experiments. *dnf1-1*, *dnf2-1*, *lin-2, ipd3-1*, 7Y (*nad1-3*), N5896 (*nad1-4*) mutants listed in Supplementary Table S1A were used for phenotypic characterization, expression analyses, and generating double symbiotic mutants. Seeds were chemically scarified with sulfuric acid and sterilized, as described in the *M. truncatula* handbook [24]. Seeds were germinated with overnight incubation in dark at room temperature on inverted agar plates (1.0% water–agar) following 4–6 days-long cold treatment at 4 °C. All of the seedlings were grown in zeolite substrate (Geoproduct Kft., Mád, Hungary) for five days before inoculation with rhizobia. Genetic crossings between symbiotic mutants and genotypes were carried out according to the method described by [24]. Double mutants were confirmed by polymerase chain reaction (PCR) amplification or sequencing the PCR products (*lin-2*) of the mutant genes using the oligonucleotide primers listed in Supplementary Table S2.

Plants were infected with rhizobial strains *Sinorhizobium* (*Ensifer*) *meliloti* 1021, *Sinorhizobium* (*Ensifer*) *medicae* WSM419 carrying the pXLGD4 plasmid, expressing the *lacZ* reporter gene under the control of the *hemA* promoter or *S. medicae* WSM419 containing the pMEpTrpGFPGUS plasmid, expressing the β-glucoronidase gene and the green fluorescent protein (Supplementary Table S1B). The pMEpTrpGFPGUS plasmid [25] was transferred into *S. mediace* by triparental mating [26]. The *S. meliloti* 1021 *nodA* mutant was created by phage transduction. The mutated *nodA* gene carrying a *Tn5* insertion was transduced from the *S. meliloti* 2011 *nodA* mutant strain (GMI5382) [27] to *S. meliloti* 1021 background using the N3 bacteriophage [28]. The *exoY* and *bacA* mutant derivatives [16,29] of *S. meliloti* strain 1021 are listed in Supplementary Table S1B. Rhizobial strains were grown in Tryptone Yeast (TY) medium that was supplemented with 6 mM CaCl_2_ [30] and with the appropriate antibiotics for 24–48 h at 30 °C, and inoculations were carried out as described by [30,31]. In hairy root transformation experiments and transient gene expression studies, *Agrobacterium rhizogenes* ARqua1 strain and *Agrobacterium tumefaciens* strain C58C1 were used.

### 2.2. DNA Isolation and Genetic Mapping

For genetic mapping and co-segregation analysis, the individuals of the *nad1-3* × A20 F2 segregating and *nad1-4* × 2HA back-crossed populations were grown in zeolite substrate, inoculated with *S. medicae* strain WSM419 (pXLGD4). F2 plants were scored for symbiotic phenotype and showing the symptoms of nitrogen starvation (presence of brown pigmentation in nodules, reduced growth habit and yellowish leaves) at 14, 21 and 28 days post-inoculation (dpi). The genomic DNA from *M. truncatula* plants was isolated using the ZenoGene40 plant DNA purification kit (Zenon Bio, Szeged, Hungary). The map position of *nad1-3* was identified by analyzing the genotypes of the mapping population for a genetic marker set of the *M. truncatula* genome [32,33].

### 2.3. Microscopy Analyses

For microscopy analyses, harvested nodules were fixed in 4% paraformaldehyde solution in phosphate buffered saline (PBS) (pH 7.4) [34] under vacuum for 3 × 30 s, and then kept at room temperature (RT) for 30 min. The nodules were embedded in 5% agarose gel and 70 µm longitudinal sections were prepared using Leica VT1200S vibratome (Leica Biosystems GmbH, Nussloch, Germany).

For LacZ activity, sections were stained with 0.1% XGal in PBS (pH 7.4) supplemented with 5 mM potassium ferricyanide and 5 mM potassium ferrocyanide for 1 h at 37 °C. Accumulation of phenolic compounds was detected by staining nodule sections with 0.1% toluidine blue solution in PBS buffer for 1 to 2 min at RT, or with a slightly modified protocol of potassium permanganate/methylene blue staining [13]. Briefly, nodule sections were vacuum-infiltrated for 30 min and post-fixed for 60 min in 2.5% glutaraldehyde in PBS (pH 7.4). Subsequently, they were rinsed in PBS (pH 7.4) three times for 5 min, followed by incubating in 0.04% aqueous solution of KMnO_4_ for 5 min and then rinsed in PBS (pH 7.4) for 5 min. The last steps of staining included with the incubation in 0.01% aqueous solution of methylene blue for 2 min, and then the nodule sections were cleared with 2.4% sodium hypochlorite for 5 min, rinsed in PBS (pH 7.4) three times for 1 min. For the detection of rhizobia expressing the β-glucuronidase gene, nodule sections were stained for 1 h at 37 °C with 2 mM X-Gluc (Duchefa Biochemie, Haarlem, The Netherlands) in PBS that was supplemented with 5 mM potassium ferricyanide and 5 mM potassium ferrocyanide.

The stained sections were observed by Olympus BX41M light microscope with 10× and 20× objectives and images were captured using an Olympus Camedia E-10 digital camera (Olympus Life Science Europa GmbH, Hamburg, Germany). For promoter analysis, 80 µm sections of nodules from transformed roots were stained with 1 mM Magenta-gluc (Duchefa Biochemie) in PBS supplemented with 5 mM potassium ferricyanide and 5 mM potassium ferrocyanide under vacuum for 30 min and 37 °C for 4–16 h. After fixation with 1.5% glutaraldehyde (Sigma, St. Louis, MO, USA), sections were washed and stained for LacZ activity as described above.

The viability test of bacteria was carried out for 20 min at room temperature on non-pre-fixed fresh nodule sections using the LIVE/DEAD BacLight™ Viability kit (Life Technologies, Carlsbad, CA, USA) containing 5 µM SYTO9 and 30 µM propidium iodide (PI) in 50 mM Tris (pH 7.0) buffer, as described in [8]. Following rinses in deionized water, the images of nodule sections were acquired with Olympus Fluoview FV1000 confocal laser scanning microscopy (Olympus Life Science Europa GmbH).

To analyze the bacteroid morphology, nodule occupancy by rhizobia and autofluorescence, nodule sections were stained in PBS (pH 7.4) containing 5 μM SYTO13 (Life Technologies) for 20 min, rinsed with 1× PBS, and sections were imaged using the same settings of confocal laser scanning microscopy, as described before [35].

To follow the subcellular localization of NAD1 and subcellular protein markers, *N. benthamiana* epidermal cells were analyzed using a Leica confocal microscope equipped with Argon (488 nm laserline) and helium–neon (543 nm laserline) lasers (Leica Microsystems, Heidelberg, Germany). Green fluorescent protein (GFP) fluorescence was imaged using excitation at 488 nm and detection between 500–550 nm. *Discosoma* sp. red fluorescent protein (DsRed)/mCherry fluorescence was imaged using excitation at 543 nm and detection between 560–650 nm.

### 2.4. Transmission and Scanning Electron Microscopy Analysis of the nad1-3 Mutant

For transmission electron microscopy (TEM), 18-day-old halved nodules fixed in 2.5% glutaraldehyde in 0.1 M phosphate buffer (pH 7.2) for 3 h were washed in a 0.1 M phosphate buffer and were post-fixed for 2 h with 1% osmium tetroxide dissolved in the same buffer. Samples were rinsed in the buffer and dehydrated in a graded ethanol series and propylene oxide and subsequently infiltrated with and embedded in Durcupan epoxy resin. Ultrathin sections (70 nm) were cut with a Reichert Jung Ultracut E ultramicrotome (Reichert-Jung Inc., Vienna, Austria) and stained with uranyl acetate (6 min) and lead citrate (6 min). Sections were viewed at 75 kV using a Hitachi 7100 transmission electron microscope (Hitachi, Tokyo, Japan).

For scanning electron microscopy (SEM), 8-day-old nodules were fixed with 2.5% glutaraldehyde in cacodylate buffer (0.05 M, pH 7.2) overnight. The next day, following 0.1% osmium tetroxide treatment (1 h, 4 °C, dark) nodules were embedded in 5% agarose gel for sectioning. The 80–100 µm sections were dehydrated in a graded ethanol series and dried with CO_2_ in a critical point dryer, followed by 10 nm of gold coating, and observed by SEM (JEOL JSM-7100F/LV; JEOL Europe BV, Zaventem, Belgium) [36].

### 2.5. Gene Expression Analysis

For real-time reverse transcription quantitative polymerase chain reaction (RT-qPCR), nodules were harvested at different time points in liquid nitrogen, RNA was extracted with TRI Reagent (Sigma) and Direct-zol RNA MiniPrep Kit (Zymo Research, Irvine, CA, USA). RNA was treated on column with DNaseI according to the manufacturer’s instructions. Total RNA was quantified using a spectrophotometer (Nanodrop-1000; NanoDrop Technologies, Wilmington, DE, USA) and checked for quality by gel electrophoresis. Complementary DNA (cDNA) was prepared from 1 µg total RNA with SuperScript III First-Strand Synthesis System for RT-PCR (Life Technologies) using oligo-dT primers according to the manufacturer’s instructions. Quantitative real time RT-PCR was performed using a LightCycler 96 (Roche, Basel, Switzerland) and qPCRBIO SyGreen Mix (PCR BIOSYSTEMS, London, UK), according to the manufacturer’s protocol. Cycle threshold values were obtained and data were analyzed by the LightCycler 96 SW1.1 software (Roche). The relative expression levels were calculated by normalization against the expression of the *PTB* (*Polypyrimidine Tract-Binding-like protein*, *Medtr3g090960*) and the ubiquitin-like gene (*Medtr3g091400*) (see Table S2 and [37]). Values of relative transcript levels were the mean of three biological replicates. Fold induction was calculated by normalizing the data for each time series to the samples of wild-type 0 dpi (time course experiment), to mock inoculated wild-type 14 dpi (experiments with *S. meliloti* 1021 mutants) and to the *M. truncatula* wild-type samples (expression analyses in plant mutants). Primers that were used for qRT-PCR are used in previous [37,38] or this studies and listed in Supplementary Table S2. Unpaired Student’s *t*-test was carried out on our RT-qPCR data sets to determine statistical significance.

### 2.6. Generating Constructs and Complementation Experiments Accomplished by Agrobacterium rhizogenes Transformation

Constructs for transformation and subcellular localization experiments were generated with the help of the Gateway Technology (Life Technologies). Fragments were amplified with Phusion DNA Polymerase (Thermo Fisher Scientific, Carlsbad, CA, USA) using mth2-62p5 BAC clone as a template and was cloned into pDONR201 vector by homologue recombination. After verification of the sequences, destination clones were generated by LR clonase II-mediated recombination. The destination vectors used were pKGW-R (complementation experiments), pKGWFS7-RR (promoter- β-glucuronidase (GUS) assay) pK7WGF2-RR and derivative with -pEF1α, pK7FWG2-RR, and pK7FWG2Δp35S (GFP or myc-tag experiments). Destination vectors that were modified and used for cloning were obtained from Plant Systems Biology, VIB-Ghent University, in which the DsRed transformation marker was cloned.

To test the complementation capacity of *NAD1* and its deletion or truncated derivatives, constructs with the complete *NAD1* gene (exon1, intron and exon2) with or without the presumed 78 base pairs (bp) intron in exon1 and only exon1 of NAD1 with or without the presumed 78 bp intron in exon1 were prepared. The *NAD1* and its mutant derivatives were expressed from a 410 bp native promoter and terminated by the 400 bp long UTR region of *NAD1*. Constructs were introduced into ARqua1 strain of *A. rhizogenes* and used for hairy root transformation, as described by [39]. The vectors contained the DsRed reporter gene to identify transgenic hairy roots. Transformed plants were planted into zeolite substrate and inoculated with *S. medicae* WSM419 (pXLGD4). Following 3–5 weeks post inoculation, nodules were collected, fixed, sectioned, and stained for LacZ activity.

For subcellular localization experiments, DNA fragments were generated by PCR on *M. truncatula* A17 genomic DNA or cDNA templates using Pfu Ultra II Fusion DNA Polymerase (Stratagene, San Diego, USA) and gene-specific primers. To generate entry clones, PCR fragments were introduced into pCR8-GW-TOPO vector (Life Technologies). To resolve the subcellular localization of the NAD1 protein, the NAD1 cDNA was recombined from the pCR8GW-TOPO entry clone into the pK7WGF2-R and pK7FWG2-R vectors carrying the AtUBQ10::DsRED1 fluorescent marker gene [40]. For co-localization experiments, p35S::GFP-NAD1 fusion was created in the pMDC43 vector [41].

### 2.7. Transient Gene Expression in Nicotiana benthamiana Epidermal Cells

Plasmid constructs were electroporated into *A. tumefaciens* strain, C58C1. Transformant agrobacteria carrying plasmids were selected on Luria-Bertani (LB) [42] medium containing appropriate antibiotics at 28 °C. A single colony was inoculated to 5 mL of LB medium, and the culture was grown at 28 °C in a shaker for 48 h. The cells were transferred to fresh LB medium containing 10 mM MES (2-morpholinoethanesulfonic acid; pH 5.6) and 40 mM acetosyringone (1:100 ratio, *v*/*v*). After 16 h of growth at 28 °C, or when the culture reached an OD600 of 3.0, the bacterial cells were collected by centrifugation at 3200× *g* for 10 min. The pellet was resuspended in 10 mM MgCl_2_ at a final optical density at λ = 600 nm (OD600) of 1.5. For *A. tumefaciens* strain p19, a final OD600 of 1.0 was used instead. Acetosyringone at a final concentration of 200 mM was added to the *Agrobacterium* solution, which was kept at room temperature for at least 3 h without shaking. For co-infiltration, an equal volume of different *Agrobacterium* strains carrying plasmids was mixed prior to infiltration. Infiltration of leaves with *Agrobacterium* cells was conducted by slowly depressing the plunger of a 1 mL disposable syringe to the surface of fully expanded leaves.

## 3. Results and Discussion

### 3.1. Mutants Defective in Symbiotic Nitrogen Fixation and Showing Induced Defense Responses Are Deficient in the NAD1 Gene

**Figure 1:**
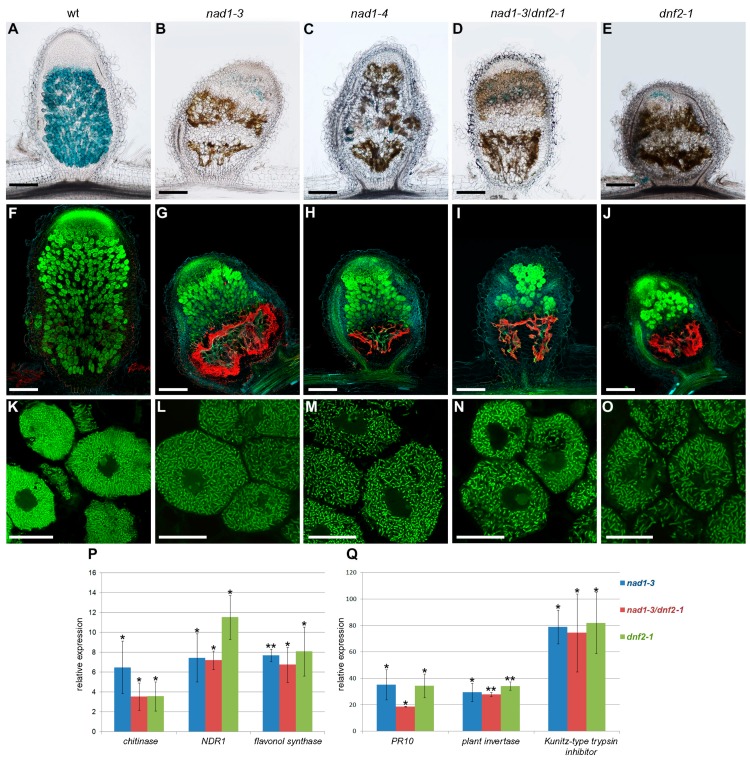
Induction of defense responses in *nodules with activated defense 1* (*nad1*), *defective in nitrogen fixation 2* (*dnf2*) and *nad1/dnf2* mutant nodules. The degree of rhizobial infection, bacteroid differentiation and the presence of brown pigmentation showing autofluoescence in symbiotically ineffective *Medicago truncatula* mutants supposed to be deficient in suppression of plant defense responses during the symbiotic interaction. Nodules were harvested 14 days post-inoculation (dpi) with *Sinorhizobium medicae* WSM419 expressing the *lacZ* marker gene. Nodule sections were stained for β-galactosidase activity (**A**–**E**) or stained with SYTO13 (**F**–**O**) and analyzed by light or confocal microscopy, respectively. Wild-type nodules showed the characteristic zonation of indeterminate nodules (**A**,**F**). Nodules of *nad1-3* (**B**,**G**), *nad1-4* (**C**,**H**), *nad1-3/dnf2* (**D**,**I**), and *dnf2* (**E**,**J**) displayed extensive brown pigmentation that corresponded to the area showing autofluorescence which is pseudocolored in red. Higher magnification revealed elongated bacteroids in the infected cells of the interzone of wild-type nodules (**K**). Elongated bacteroids were detected in the last layers of infected cells in mutant nodules (**L**,**M**,**N**,**O**), which indicates the initiation of bacteroid development in these nodules. Scale bars: (**A**–**J**) 200 μm, (**K**–**O**) 20 μm. (**P**,**Q**) Transcriptional activation of defense-related genes in the nodules of *nad1-3, nad1-3/dnf2* and *dnf2* mutants. The expression level of a *chitinase*, the *NDR1*, *flavonol synthase*, the *PR10*, a plant *invertase* and a *Kunitz-type trypsin inhibitor* gene was identified by quantitative reverse transcription PCR 14 dpi with *S. medicae* WSM419. The transcript levels were identified using three biological replicates and calculated relative to the expression detected in wild-type nodule. Error bars indicate ± standard error (SE). ** and *, *p* ≤ 0.01 and 0.05, respectively.

A collection of *M. truncatula* mutants showing defects in symbiotic nodule development and function was identified and described previously [35]. One of the ineffective mutants in this mutant collection, termed 7Y, showed the symptoms of nitrogen starvation and developed slightly cylindrical yellowish or brownish nodules following inoculation with compatible rhizobia ([35] and Supplemenatary Figure S1). The brown pigmentation associated with strong autofluorescence that appeared after inoculation suggested the induction of defense responses in 7Y mutant nodules ([35] and Figure 1B,G). Based on genetic and sequence analyses described below, another allele of 7Y, the N5896A mutant showing similar nodulation phenotype (Figure 1C,H), was identified in a separate symbiotic screen of a *M. truncatula Tnt1/MERE1* insertion mutant collection established in the Jemalong 2HA background [43,44].

Our previous study [35] revealed that 7Y is not an allele of the previously described *dnf* symbiotic mutants [45], and identified the map position of the 7Y mutant locus on the upper arm of chromosome 7 of *M. truncatula* between the genetic markers MtB243 and MtB183 ([33] and Figure S2A). A map-based cloning approach was applied to identify the gene affected in the *nad1-3* mutant. An extended segregating population of 727 F2 individuals was used to map the mutant locus between genetic markers EF4142291 and h2_96b16t19 (Figure S2A). The analysis of the sequence of this genomic region of 153 kb revealed 24 predicted gene models (Mt4.0 JBrowse) [46] including the nodule specific *NAD1* gene (*Medtr7g022640*) [21], providing a good candidate for the mutated gene in 7Y.

**Figure 2:**
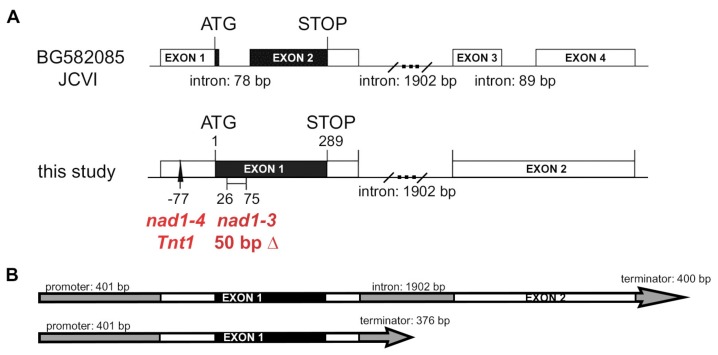
Gene structure of *NAD1* supported by experimental data and position of the mutations in *nad1-3* and *nad1-4* mutants. (**A**) The gene structure of *NAD1* proposed based on expressed sequence tag BG582085 at JCVI (upper image) and the cDNA sequence identified in this study. Black boxes represent the coding sequences while blank boxes show non-coding exons. The 50-bp deletion in *nad1-3* and the *Tnt1* insertion in *nad1-4* are shown on the cDNA-based gene structure. (**B**) The schematic presentation of the two constructs used in complementation experiments. The longer construct could completely, while the shorter one could only partially restore the symbiotic phenotype of *nad1-3* and *nad1-4* mutants (Supplementary Figure S3). Grey bars represent promoter and intron sequences, grey arrows show the terminator regions. Blank bars present exons, while black regions show the coding sequence of *NAD1*.

Oligonucleotide primers were synthesized for the *NAD1* gene and PCR reactions were carried out to amplify genomic fragments of *NAD1* from the 7Y mutant. The sequence analysis revealed a 50-bp deletion starting at 26 bp downstream of the predicted AUG start codon in 7Y; therefore, 7Y is hereafter termed *nad1-3* (Figure 2A). The gene structure of *NAD1* available at the Medicago genome database (Mt4.0 JBrowse) is based on an expressed sequence tag (EST) sequence (EST483823; GenBank BG582085.1), predicting that the *NAD1* gene consists of four exons encoding a 70 amino acid long putative protein (Mt4.0 JBrowse and Figure 2A). Because the 50-bp deletion is located in the first predicted intron of this *NAD1* gene model, cDNA of *NAD1* was generated and sequenced to search for other transcript versions. The sequence analysis revealed that the gene is actually composed of two exons and one intron (Supplemenatary Figure S2A). A protein of 96 amino acid residues with two transmembrane domains is encoded in the first exon, as described by [21]. Based on this gene structure, the 50-bp deletion in *nad1-3* mutant is located in the first exon of *NAD1* (Figure 2A). This deletion generates a frameshift and a premature translation termination in the position 202 bp of the coding sequence, and, thus, a truncated and inaccurate protein with non-NAD1-specific amino acids from the position of the ninth residues.

To identify which *Tnt1/MERE1* insertion caused the deficiency in the ineffective symbiotic mutant N5896A, the co-segregation of flanking sequence tags (FSTs) and the mutant phenotype—nitrogen deficiency and accumulation of brownish pigmentation in the nodules—was analyzed in an F2 population generated by self-pollinating an F1 plant originating from a back-cross. The genetic analysis of 40 F2 plants, including seven homozygote mutants, revealed co-segregation of the mutant phenotype and the presence of a *Tnt1* retroelement inserted into the 5′-untranslated region (UTR) of the *NAD1* gene (Figure 2A), indicating that this ineffective mutant carries an additional allele of *nad1;* therefore, N5896A ineffective mutant hereafter termed *nad1-4*. To analyze the effect of the *Tnt1* insertion in the 5′-UTR on the expression of *NAD1*, the cDNA samples that were prepared from *nad1-4* nodules were used in RT-PCR which showed the absence of NAD1 transcript indicating that the *Tnt1* insertion in *nad1-4* abolished the expression and activity of *NAD1* (Supplementary Figure S2B).

### 3.2. Both Exons of NAD1 Are Required for Complete Rescue of the Symbiotic Phenotype of nad1-3 and nad1-4 Mutants

In order to test whether the entire transcript of *NAD1* harboring two exons or other alternative transcripts are able to restore the symbiotic phenotype of *nad1-3* and *nad1-4*, we carried out genetic complementation experiments using the following constructs: the genomic copy of *NAD1*, the first exon (containing the entire coding sequence for the protein), the first exon with a 78-bp deletion mimicking the first intron predicted at the Medicago genome database based on the BG582085 EST and the whole gene with the same 78-bp deletion fused to the native *NAD1* promoter (Figure S3). These constructs were introduced into *nad1-3* and *nad1-4* roots using *Agrobacterium rhizogenes* mediated hairy root transformation. The roots were inoculated with *S. medicae* strain WSM419 (pXLGD4), and the symbiotic phenotype was assessed for the presence of infected cells in the nodules with the help of X-gal staining, as well as for the vanishing of the brown pigmentation, the hallmark of the *nad1* mutation. Brownish empty nodules were only formed on the *nad1-3* roots transformed either with the empty vector (Supplementary Figure S3D–F) or the constructs harboring the 78-bp deletion that mimics the first intron prediction at the *Medicago* genome database, based on the BG582085 EST sequence (Supplementary Figure S3L,N). This result proved that the lack of this sequence presumed to be the “first intron” abolishes the function of NAD1, so it is an integral part of the first exon. The mixture of wild-type nodules with proper zonation (Supplementary Figure S3J) and nodules showing brown pigmentation (Supplementary Figure S3K) developed on *nad1-3* roots transformed with the first exon harboring the coding sequence for the NAD1 protein, suggesting the loss of proper regulation of *NAD1* in the absence of the rest of the transcript and/or the intron and/or the 3′-UTR to restore the symbiotic phenotype. The nodules on *nad1* mutants transformed with the complete *NAD1* gene were pink, indicating that they were functional nodules (Supplementary Figure S3G). The X-gal stained nodules showed the typical zonation of the indeterminate nodules with invaded cells by rhizobia (Supplementary Figure S3I,M), demonstrating that the complete gene is essential for the full rescue of the symbiotic phenotype of *nad1-3*.

The results of the complementation experiments confirmed the proper structure of the *NAD1* gene, and, in addition, the requirement of the non-coding second exon for the complete capacity of *NAD1* to restore the symbiotic phenotype was demonstrated. These results point out the potential regulatory function of exon2 and/or 3′UTR of *NAD1*, which, in turn, suggests that the BG582085 sequence correspond to either an aberrant transcript or an alternative spliced product of the *NAD1* gene.

### 3.3. NAD1 Is Expressed in Infected Cells and the Gene Product Localizes to the Endoplasmic Reticulum

**Figure 3:**
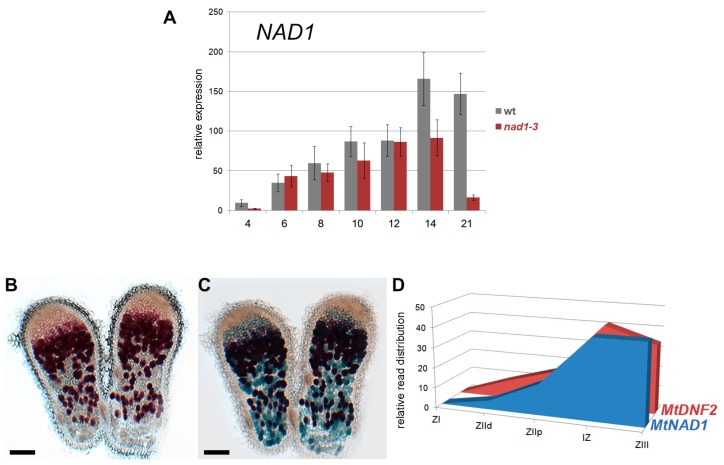
*NAD1* is induced following rhizobial inoculation and predominantly expressed in the intermediate and nitrogen fixation zones of *Medicago truncatula* nodules. (**A**) The expression level of *NAD1* during nodule development in wild-type Jemalong and *nad1-3 M. truncatula* plants inoculated with *S. medicae* WSM419 (pXLGD4). The transcript levels were identified by reverse transcription quantitative polymerase chain reaction (RT-qPCR) using three biological replicates and calculated relative to the expression detected in wild-type at 0 dpi; *x*-axis shows dpi. The results were normalized using an *UBQ-*like gene and *PTB.* Error bars indicate ± SE. (**B**–**D**) *NAD1* is expressed in the infected cells of the infection zone, the intermediate zone and the nitrogen-fixation zone of *M. truncatula* nodules. The *NAD1* promoter-β-glucuronidase (GUS) fusion reporter construct (*pNAD1::GUS)* was introduced into the roots of wild-type plants with *Agrobacterium rhizogenes* mediated hairy root transformation. Roots were inoculated with *S. medicae* WSM419 expressing the *lacZ* reporter gene. Nodules on transformed roots at 14 dpi were stained first for GUS activity using the (**B**) Magenta-Gluc substrate followed by staining for (**C**) β-galactosidase activity to display the presence of rhizobia. Scale bars: 200 μm. (**D**) Relative spatial expression of *NAD1* and *DNF2* generated with RNA sequencing of different nodule zones obtained with laser-capture microdissection described in the study by [47]. Both *NAD1* and *DNF2* are expressed in the zones of the indeterminate nodule of *M. truncatula* containing colonized cells. ZI: zone I (meristem); ZIId: invasion zone distal; ZIIp: invasion zone proximal; IZ: intermediate zone; ZIII nitrogen-fixation zone.

The expression of *NAD1* is nodule specific and its activation requires the formation of symbiotic nodules [21]. The expression of *NAD1* was monitored during the nodule development using quantitative RT-PCR. The *NAD1* is expressed at a low level at 4 dpi with rhizobia and a strong increase in expression was detected at 6 dpi, and subsequent time points in wild type nodules (Figure 3A). In *nad1-3* nodules, the *NAD1* was activated similarly, but it was expressed at a lower level when compared to wild type samples between 8 and 21 dpi (Figure 3A). We detected a great decline in the *NAD1* expression 21 dpi, which is probably associated with the advanced stage of the necrotic phenotype of *nad1-3* nodules. 

To further analyze the expression pattern of *NAD1*, its promoter fused to the β-glucuronidase reporter gene was introduced into wild-type *M. truncatula* roots using *A. rhizogenes*-mediated hairy root transformation. The roots were inoculated with *S. medicae* strain WSM419 (pXLGD4) and nodules on transformed roots were monitored for GUS activity using 5-bromo-6-chloro-3-indolyl β-D-glucopyranosiduronic acid cyclohexylammonium salt (Magenta-Gluc) substrate and then stained for β-galactosidase activity to visualize the presence of rhizobia at 14 dpi. GUS activity was found in the cells of the invasion zone, the intermediate zone, and the nitrogen fixation zone, as well (Figure 3B). All of these cells were occupied by rhizobia (Figure 3C), indicating that *NAD1* is expressed in the infected cells of the symbiotic nodule.

The observed expression pattern of *NAD1* was in agreement with RNA-sequencing (RNA-seq) data of different nodule zones obtained by laser-capture microdissection [47]. The *NAD1* gene is induced in the infection zone and reached its maximum activity in the transition between the infection and nitrogen fixation zones, and maintained in the nitrogen fixation zone (Figure 3D). Based on the RNA-seq data, the *DNF2* gene also required for the suppression of plant immunity during nitrogen fixing symbiotic interaction [19], shows co-expression with *NAD1* in the zones of *M. truncatula* nodules (Figure 3D). The expression pattern of *NAD1* in the nodule zones indicates the continuous requirement of *NAD1* in infected nodule cells during the symbiotically active lifetime of the nodule.

**Figure 4:**
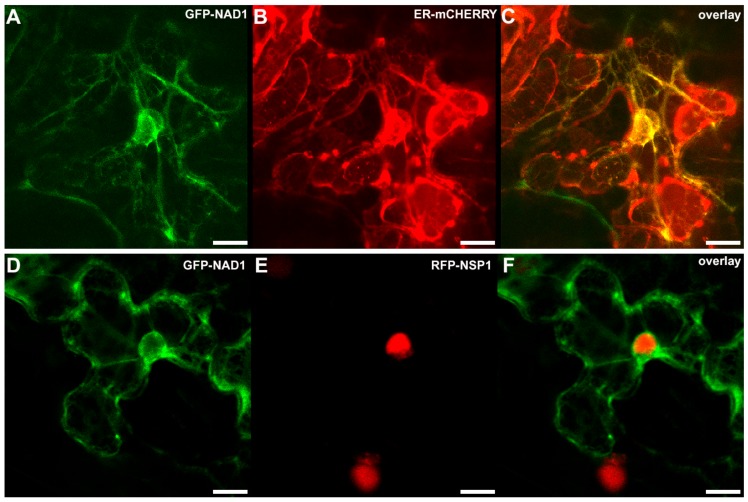
Co-localization of NAD1 with localization control constructs in *Nicotiana benthamiana* leaf epidermal cells. The *p35S::GFP-NAD1* and the ER marker *p35S::ER-mCherry* or the nucleus marker *pUbq10::RFP-NSP1* constructs were co-transformed transiently into *Nicotiana benthamiana* leaves. The localization of the tagged proteins was imaged by confocal microscopy. The signal of GFP-NAD1 was detected in the endoplasmic reticulum (ER) network (**A**,**D**), which was confirmed by the co-localization with the mCherry signal targeted to the ER (**B**). The GFP-NAD1 is excluded from the nucleus but NSP1 shows nucleus localization (**E**). Panels C and F show overlay images of A and B, D and E, respectively. Scale bars: (**A**–**I**) 10 μm.

The sub-cellular localization of the proteins might help to elucidate their functional properties. The NAD1 is predicted to have two transmembrane domains [21], suggesting to be localized to the plasma membrane and/or subcellular membrane compartment. In order to study the localization of NAD1, constructs coding for *NAD1* proteins tagged either C- or N-terminally with GFP or c-myc epitopes were created and introduced into *nad1-3* roots using hairy root transformation. Unfortunately, none of the constructs restored the symbiotic phenotype, indicating that these tags were probably interfered with the function of NAD1 (data not shown). To further investigate the subcellular localization of NAD1, the constructs of N- and C-terminal GFP fusions of the full-length *NAD1* cDNA were expressed transiently under the control of the CaMV p35S promoter in *Nicotiana benthamiana* leaves. According to the detected GFP fluorescence in the leaf epidermal cells, NAD1 was found to be associated exclusively with the endoplasmic reticulum (ER) membrane network (Figure 4). In order to confirm the localization pattern of the NAD1 protein, the p35S::GFP-NAD1 fusion was co-transformed with organelle marker constructs [48] harboring well-established targeting sequences fused to mCherry. We could not detect any significant overlap with the Golgi, tonoplast and peroxisome-specific markers (data not shown), however NAD1 co-localized with the ER-specific marker (Figure 4C). *NAD1* codes for a small protein with a 10.7 kDa molecular weight. It has been generally admitted that the diffusion limit set by the nuclear pore for protein is 60 kDa. Moreover, the nuclear localization of a GFP3 oligomer protein, whose size is around 90 kDa, was observed recently [49]. Thus, GFP fusions below this size without any localization signal, theoretically, can enter into the nucleus by passive diffusion. When considering the fact that GFP-NAD1 chimeric protein is still under this size limit (38 kDa), there was a theoretical chance that GFP carries the small NAD1 into the nucleus. To exclude this possibility, we co-expressed the p35S::GFP-NAD1 constructs with the pUbq10::RFP-NSP1 [40], showing strong nuclear localization. According to this experiments, the GFP-NAD1 fusion protein was clearly excluded from the nucleus (Figure 4F).

Our transient co-localization studies in *N. benthamiana* leaves suggesting the association of the NAD1 signal to the endoplasmic reticulum (ER) is in agreement with the previous results that were obtained from localization studies in restored functional nodules and in *Arabidopsis* protoplasts [21]. In this previous study, the flag-tagged NAD1 restored the symbiotic phenotype of *nad1* nodules, indicating that the flag-tag did not interfere with the activity of NAD1 and the more sensitive immunofluorescence assay detected the signal of NAD1 on ER. The ER localization of NAD1 proved by three independent methods supports plausibility of the association of NAD1 to the ER. The ER serves many general functions in the cell, and have a role in protein and lipid biosynthesis and transport, protein folding, signaling (calcium storage) [50], and even in immunity [51], which can inspire several theories about the actual function of NAD1 in the root nodule symbiotic interaction.

### 3.4. The Defense Responses Are Induced Simultaneously in nad1 and dnf2 Mutants

**Figure 5:**
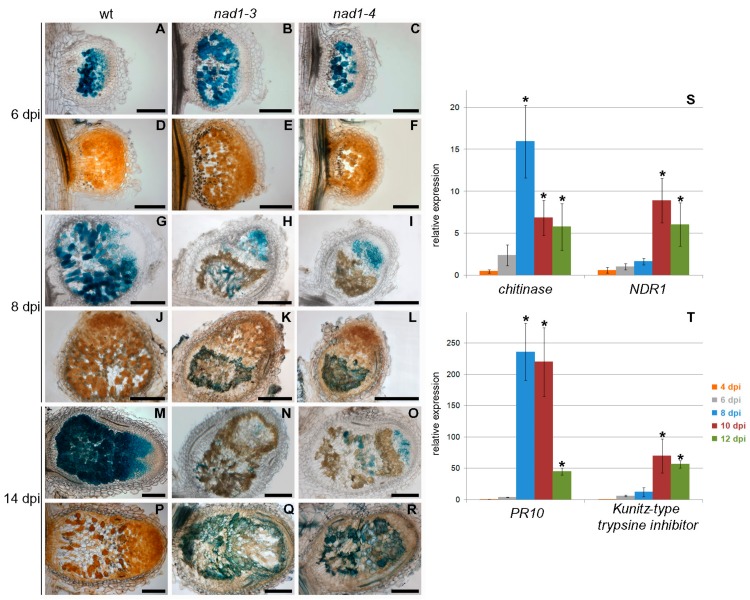
(**A**–**T**) Defense responses are activated in *nad1-3* and *nad1-4* nodules between 6 and 8 dpi with *S. medicae* WSM419 carrying the GUS reporter gene. (**A**–**R**) The brown pigmentation in *nad1* nodules corresponds to accumulation of phenolic compounds. Nodule sections of wild-type (first column) and *nad1-3* (second column) and *nad1-4* (third column) mutants were stained for the activity of β-glucuronidase (**A**–**C**,**G**–**I**,**M**–**O**) to detect the presence of bacteria and with potassium permanganate and methylene blue dye (**D**–**F**,**J**–**L**,**P**–**R**) to visualize phenolic compounds in blue at 6 (**A**–**F**), 8 (**G**–**L**) and 14 dpi (**M**–**R**). Wild-type and mutant nodules are colonized equally and no accumulation of polyphenolics was detected 6 dpi (**A**-**F**). The brown pigmentation appeared in *nad1* nodules at 8 dpi (**H**,**I**) overlaps with the region containing polyphenols (**K**,**L**), indicating the activation of defense responses in mutant nodules. The accumulation of polyphenolic compounds expanded in increased area of the mutant nodules at 14 dpi (**N**,**O**,**Q**,**R**) but brown pigmentation was not detected in wild-type nodules at any time points. Scale bars: (**A**–**R**) 200 μm. Temporal induction of defense-related genes (a *chitinase*, *NDR1*, *PR10,* and a *Kunitz-type trypsin inhibitor*) in *nad1* nodules analyzed with quantitative RT-PCR following *S. medicae* WSM419 inoculation. Relative transcript levels at different time points were calculated in relation to wild-type plants (**S**,**T**). Error bars indicate ± SE. * *p* ≤ 0.05.

**Figure 6:**
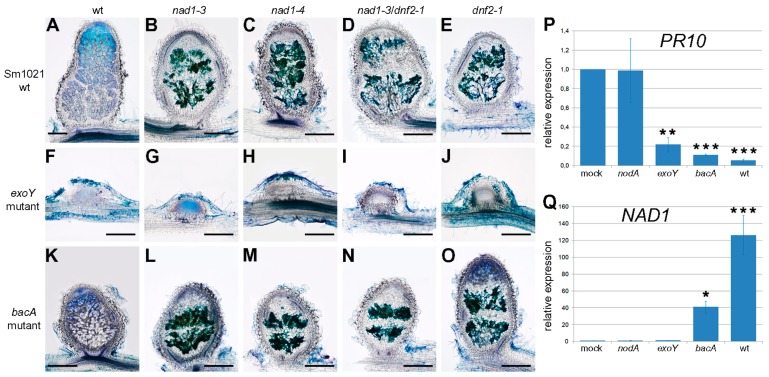
Positioning of the function of NAD1 in the symbiotic process. Sections of wild-type (**A**,**F**,**K**), *nad1-3* (**B**,**G**,**L**), *nad1-4* (**C**,**H**,**M**), *nad1-3/dnf2-1* (**D**,**I**,**N**), and *dnf2-1* (**E**,**J**,**O**) nodules were stained with toluidine blue to detect accumulation of phenolic compounds 14 dpi with wild-type *S. meliloti* 1021 (**A**–**E**) or its *exoY* (**F**–**J**) and *bacA* (**K**–**O**) mutant derivatives. Nodule primordia induced by *exoY* mutant rhizobia displayed no staining of polyphenols, but mutant nodules elicited by *S. meliloti* wild-type or *bacA* mutant stained dark green showing the accumulation of phenolic compounds which indicates that *NAD1* acts prior bacterial differentiation. Scale bars: (**A**–**O**) 200 μm. The *PR10* and *NAD1* genes are expressed inversely in nodules containing released rhizobia in 14 dpi nodules (**P**,**Q**). The relative expression level of *PR10* and *NAD1* was analyzed in wild-type (Jemalong) nodules with quantitative RT-PCR 14 dpi with wild-type and mutant *S. meliloti* 1021 derivatives blocked at different stages of the symbiotic process. The transcript levels were calculated relative to the expression detected in non-infected roots (mock). Values of relative transcript levels are the mean of three biological replicates. Error bars indicate ± SE. ***, ** and *, *p* ≤ 0.001, 0.01 and 0.05, respectively.

The accumulation of brown pigmentation that was reported in the nodules of the *M. truncatula dnf2* mutant [19] was similar to those detected in *nad1-3* and *nad1-4* nodules (Figure 1). To characterize in more details and discriminate them if possible, the progression of the rhizobial infection in the nodules was analyzed and defense responses were compared in these and in generated double mutants. For this, longitudinal sections of wild-type, *nad1-3, nad1-4*, *dnf2-1*, and *nad1-3*/*dnf2-1* double mutant nodules at 14 dpi with *Sinorhizobium medicae* WSM419 (pXLGD4) constitutively expressing the *lacZ* gene were stained for β-galactosidase activity and were investigated by light and fluorescence microscopy. Wild-type nodules showed the typical zonation of indeterminate nodules with fully infected cells in the nitrogen fixation zone (Figure 1A), but *nad1*, *dnf2,* and *nad1/dnf2* mutant nodules did not show strong β-galactosidase activity, indicating the low occupancy of rhizobia in nodule cells (Figure 1B–E). The nodulation phenotype of the single *nad1*, *dnf2,* and the *nad1/dnf2* double mutants were indistinguishable, indicating that the two mutations affect the same or very similar pathways. Staining with X-gal did not clearly reveal the zonation of the mutant nodules, therefore, they were further analyzed by confocal laser scanning microscopy using the nucleic acid-binding dye SYTO13 [52]. Wild-type and mutant nodules did not show differences in the infection zone and the transition between the infection and nitrogen fixation zones (Figure 1F–J). In wild-type nodules, differentiated bacteria in the interzone were oriented toward the vacuoles (Figure 1K). In contrast, in the last layers of infected cell in the interzone of mutant nodules, rhizobia were disordered and slightly elongated (Figure 1L–O). The mutant nodules in the fixation zone were devoid of bacteria (Figure 1G–J). Moreover this region displayed strong autofluorescence in all of the mutant nodules (Figure 1G–J), as described previously for *nad1* nodules [21,35], suggesting the accumulation of phenolic compounds. The presence of phenylpropanoids was confirmed with the potassium permanganate/methylene blue staining procedure that reveals polyphenolics with blue coloration and with the toluidine blue dye which stained phenolic compounds dark greenish (Figure 5J–L,P–R and Figure 6A–E). The deposition of polymeric phenols is considered as a defense response [53], and, therefore, the induction (or lack of suppression) of defense responses can be anticipated in *nad1*, *dnf2,* and *nad1/dnf2* mutant nodules. To assess the activation of plant defense responses in *nad1-3*, *dnf2,* and *nad1-3/dnf2* nodules, we monitored the transcriptional activation of defense-related marker genes at 14 dpi with *S. medicae* strain WSM419 using quantitative RT-PCR. A *chitinase* (*Medtr3g118390*), the *NDR1* (a Non-race-specific Disease Resistance, *Medtr5g076170*), a *flavonol synthase* (*Medtr5g055680*), a *PR10* (*Medtr2g035150*), a *plant invertase* (*Medtr4g101760*), and a *Kunitz-type trypsin inhibitor* (*Medtr6g078250*) defense-related genes were up-regulated in the *nad1-3*, *dnf2-1,* and *nad1-3/dnf2-1* double mutant nodules (Figure 1P,Q), confirming induced defense responses, similarly, as it was found previously in the *M. truncatula dnf2* [19] and *symcrk* [20] mutants.

As commented before, microscopy studies revealed that nodules of *nad1-3* and *nad1-4* mutants showed brown pigmentation that fluoresced, indicating the accumulation of phenolic compounds at 14 dpi (Figure 1). To define at what stages of the symbiotic nodule development the defense responses are activated, the time course of phenolic compound accumulations in nodules was analyzed using potassium permanganate/methylene blue staining. In wild-type Jemalong nodules, cells were colonized by bacteria in the infection, inter and nitrogen fixation zones and no polyphenol accumulation could be detected at any time point of the analysis (Figure 5A,D,G,J,M,P). In the mutant nodules, no polyphenolic staining was observed at 6 dpi (Figure 5E,F). Blue precipitates, indicating that the presence of polyphenolics could be detected at 8 and 14 dpi that correspond to the sites of brown pigmentation (Figure 5K,L,Q,R). Consistent with the accumulation of phenolic compounds, the expression of defense-related genes were strongly activated at 8 (the *chitinase* and *PR10*) and 10 dpi (*NDR1* and the *Kunitz-type trypsin inhibitor*) in *nad1-3* mutant nodules, relative to wild-type (Figure 5S,T).

To reveal any differences in the induction of plant defense responses, the kinetics of the appearance of brown pigmentation was analyzed in *nad1-3*, *dnf2-1* and *nad1-3/dnf2-1* double mutants inoculated with *S. meliloti* strain WSM419 (pXLGD4). Nodules were stained for β-galactosidase activity and the microscopy analysis revealed invaded nodule cells in the mutants at 6 dpi and the brown pigmentation appeared at 8dpi in *dnf2-1* and *nad1-3/dnf2-1* nodules, similar to *nad1-3* (Supplementary Figure S4H,M,R). These results indicate that *nad1* and *dnf2* mutant nodules not only show comparable induction of defense-like responses but these reactions exhibit similar kinetics, as well (Figure 5 and Supplementary Figure S4).

### 3.5. Defense-Like Responses Are Induced upon the Internalization of the Symbiotic Nodule Cells by Rhizobia and Rapidly Induce the Degradation of Host and Bacterial Cells

Because the defense responses were induced between 6 and 8 dpi in *nad1* and *dnf2* nodules, we analyzed the viability of rhizobia using live and dead staining with the fluorescent nucleic acid-binding dye, SYTO9 and PI using confocal microscopy. SYTO9 detects living bacteria showing green fluorescence while plant nuclei and membrane compromised bacterial cells take up PI and show red fluorescence. At 6 dpi, viable green fluorescent bacteria were detected in wild-type and *nad1-3*, *dnf2-1* and *nad1-3/dnf2-1* double mutant nodules and only the meristematic region composed of non-infected small cells rich in cytoplasm as well as plant nuclei displayed red fluorescence (Supplementary Figure S5B,C,H,I,N,O,T,U). In the sections of 8 dpi wild-type nodules, bacteria fluoresced green (Supplementary Figure S5E,F). In mutant nodules, a large area with autofluorescence appeared few layers below the invaded cells and only few cells containing dead red fluorescent bacteria could be observed in the transition zone between the invaded cells and the area accumulating phenolic compounds, suggesting the rapid disintegration of dead bacteria in nodule cells (Supplementary Figure S5K,L,Q,R,W,X).

To further analyze the induced defense responses in *nad1-3* nodules at ultrastructural level, electron microscopy studies on 8 and 18 dpi nodules were carried out. The SEM analysis of developing 8 dpi wild-type nodules detected elongated bacteroids that were orientated towards the central vacuoles and were encompassed by cytoplasmic matrix in the cells of the first layers of the nitrogen fixation zone (Supplementary Figure S6B,C). The nodule cells in the corresponding region in *nad1-3* nodules have thickened cell walls and they were either devoid of rhizobia or contained slightly elongated, disordered, and aggregated bacteria without surrounding cytoplasmic matrix, indicating the degradation of bacterial and plant cells (Supplementary Figure S6E,F). Transmission electron microscopy images showed differentiated bacteroids, reaching 6–8 μm in length, in the nitrogen fixation zone of 18 dpi wild-type nodules (Supplementary Figure S6G,J). In *nad1-3* nodules, cells in the last layers of infected cells had thickened cell walls and often cell wall-like deposits surrounding electron dense necrotic bacteria were detected (Supplementary Figure S6H,I). In root distal part of the *nad1-3* nodules corresponding to zone III, disorganized cellular structure and hydrolyzed cell wall remnants were identified, indicating the necrosis of both the host cells and bacteroids (Supplementary Figure S6K). Older cells in the proximal part of the nodules were almost empty, showing the advanced degradation of symbiotic cells in *nad1-3* nodules (Supplementary Figure S6L).

In our experimental conditions, the cells in the invasion and the first layer of the intermediate zone of the indeterminate *M. truncatula* nodules were invaded by rhizobia at 6 dpi, but no visible signs of induced defense response or induction of defense-related genes was observed in *nad1* nodules (Figure 5, Supplementary Figures S4 and S5). In contrast, the viability staining of bacteroids, the appearance of accumulated polyphenolics and the activation of defense-associated genes suggested the loss of the control over the plant immune responses in *nad1* nodules at 8 dpi. Moreover, electron micrographs of *nad1-3* showed the rapid death of rhizobia and the lysis of the host cell resulting in almost empty cells in the proximal part of the nodule, indicating the quick progression of necrosis in 8 dpi *nad1* nodules (Supplementary Figure S6). Our microscopy analyses highlighted that bacteria not only filled the host cells in the transition zone between invasion and nitrogen fixation zone, but their differentiation was initiated in *nad1* nodules.

### 3.6. The Function of NAD1 Precedes DNF1 and NAD1 Acts Independently and Prior to Rhizobial BacA in the Symbiotic Process

To further explore at what stage the symbiotic interaction is blocked, the nodulation phenotypes of *nad1-3*, *nad1-4*, *nad1-3/dnf1-1*, *nad1-3/dnf2-1* and *nad1-3/lin-2* mutant plants were analyzed after inoculation with rhizobial strains deficient in the production of the NF (*nodA*) or the succinoglycan EPS I (*exoY*) or defective in bacteroid differentiation (*bacA*). To assess the induction of plant defense responses in wild-type and mutant nodules that were arrested at different stages of the symbiotic interaction, sections of the nodules were stained with toluidine blue and analyzed for the presence of phenolic compounds at 14 dpi. Inoculation of the symbiotically less effective rhizobial strain *S. meliloti* 1021 [54] caused a similar accumulation of polyphenolics in *nad1*, *dnf2,* and *nad1/dnf2* nodules (Figure 6A–E) that was found with *S. medicae* WSM 419, indicating that the *nad1* phenotype is not strain dependent. This allowed for us to analyze the symbiotic phenotype of wild-type and *nad1-3, nad1-4, dnf2-1,* and *nad1-3/dnf2-1* mutants that were inoculated with symbiotic bacterial mutants generated in the *S. meliloti* 1021 strain.

The *exoY* mutant of *S. meliloti* strain 1021 defective in the production of succinoglycan fails to initiate infection thread formation, hence ineffective nodules without rhizobial invasion are formed on *Medicago* roots ([55] and Figure 6F). In mutant nodules, no accumulation of the phenolic compounds could be observed at 14 dpi, suggesting the requirement of bacterial colonization for the induction of plant defense in these symbiotic mutants (Figure 6F–J).

The BacA protein protects *S. meliloti* against the antimicrobial activity of NCR peptides [8], and, therefore, BacA is essential for bacteroid development in galegoid legumes [56]. *bacA* mutants induce indeterminate nodule formation on *Medicago* roots but rhizobia are killed soon after release from infection threads prior to bacteroid differentiation [57]. Accordingly, *bacA* mutant rhizobia lysed and non-infected cells were observed in wild-type Jemalong nodules (Figure 6K). This inefficient interaction does not trigger phenolic compounds production [23]. In contrast, the nodules of *nad1, dnf2-1,* and *nad1-3/dnf2-1* mutants that were elicited by *bacA* show accumulation of phenolic compounds, indicating that the defense responses were rapidly activated following the release of mutant rhizobia into the host compartment (Figure 6L–O).

To correlate the induction of *NAD1* and plant defense responses in nodules elicited by wild-type and symbiotically deficient rhizobial strains, the transcriptional activation of *NAD1* and the defense-related *PR10* was monitored in wild-type Jemalong roots or nodulated roots at 14 dpi. The basic expression level of the *PR10* gene detected in nodules elicited by mock or nodulation factor (NF) deficient *S. meliloti* [58] was suppressed following inoculation with wild-type rhizobia or bacteria defective in later stages of the symbiotic process, suggesting the suppression of *PR10* upon the initiation of infection thread development (Figure 6P). The induction of the *NAD1* expression following the inoculation with *bacA* mutant and wild-type *S. meliloti* but not with *S. meliloti nodA* and *exoY* mutants indicated the need of bacterial release for the induction of *NAD1*. These results showed that NAD1 starts to function after the uptake of rhizobia into the host cells but prior rhizobial differentiation in *M. truncatula* nodules.

**Figure 7:**
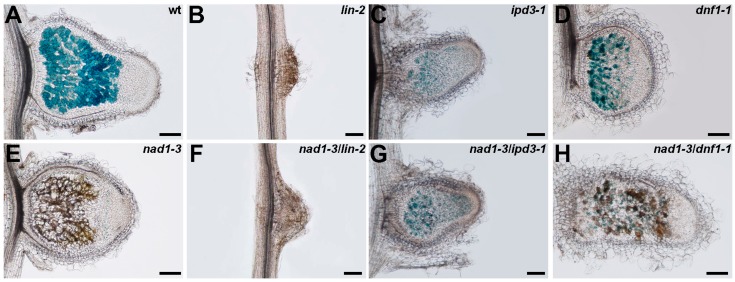
The defense responses are activated in *nad1-3* following the internalization of rhizobia but prior to bacteroid differentiation. Nodulation phenotype of wild-type (Jemalong) (**A**), *lin-2* (**B**), *ipd3-1* (**C**), *dnf1-1* (**D**), *nad1-3* (**E**), *nad1-3/lin-2* (**F**), *nad1-3/ipd3-1* (**G**), and *nad1-3/dnf1-1* (**H**), mutants 14 dpi with *S. medicae* expressing the *lacZ* marker gene. The induction of defense response was detected by the presence of natural brown pigmentation (no staining) in mutant nodules. Scale bars: 200 μm

The characterization of the mutant phenotype of *nad1* and other ineffective symbiotic nodulation double mutants with *nad1* enables the determination of the functional hierarchy of the impaired genes. The symbiotic phenotype of single and double mutants of *nad1-3*, *lin-2*, *ipd3-1,* and *dnf1-1* were analyzed at 14 dpi with *S. medicae* (pXLGD4). The *lin-2* mutant is deficient in the early stage of the rhizobial symbiotic process and although nodule primordia are formed, the infection thread development is arrested in the root hairs ([59] and Figure 7B). The nodules on *ipd3* mutant roots are more developed when compared to *lin* nodule primordia, but *ipd3* nodules are impaired in release of rhizobia from infection threads, and, thus, bacteria do not colonize nodule cells ([60] and Figure S7C). The *nad1-3/lin-2* and *nad1-3/ipd3-1* double mutants showed the same defects of infection and bacterial release as *lin-2* and *ipd3-1*, respectively (Figure 7F,G), without phenolics accumulation, indicating that bacterial release and invasion is required for the function of *NAD1*.

The *DNF1* gene coding for a symbiosis-specific component of the secretory pathway is required for bacterial differentiation and symbiosome development and in *dnf1* mutant, released bacteria are blocked in the very early stage of bacteroid development [6,7]. In the undeveloped nodules of *dnf1,* no distinct developmental zones could be observed, but nodule cells were colonized by rhizobia with no accumulation of phenolic compounds (Figure 7D). The presence of phenolic compounds in the *nad1-3/dnf1-1* double mutants suggests the activation of plant defense responses (Figure 7H). This was consistent with our previous findings that the proper acting of NAD1 is required before bacterial differentiation.

Our microscopy analysis of the nodulation phenotypes of single and double ineffective plant symbiotic mutants that were inoculated with wild-type and symbiotic mutant rhizobia allowed for us to position the *NAD1* gene in the symbiotic process. First of all, the nodulation phenotype of *nad1* and *nad1-3/ipd3-1* double mutants that were inoculated with *exoY* mutant and wild-type rhizobia, respectively, indicated that bacterial release and colonization of nodule cells are required for the function of NAD1. The observation that *dnf2*, *nad1,* and *nad1/dnf2* nodules, as elicited by *bacA* mutant rhizobia, did accumulate phenolic compounds suggested that the repression of plant defense responses is independent of the differentiation of rhizobia at this stage of the interaction. The symbiotic interaction is blocked at the same developmental stage and the expression of the defense-associated genes displayed the similar induction in *nad1*/*dnf2* double mutant as in the single mutant nodules, suggesting that *NAD1* and *DNF2* operate close together in the same developmental pathway. The observation that the plant defense is suppressed irrespective of the differential status of bacteria was further evidenced by the microscopy analysis of the *nad1-3/ dnf1-1* double mutant that was elicited with wild-type bacteria. We detected brown pigmentation in *nad1-3/dnf1-1* double mutant nodules, although this defense reaction was not as robust as in *nad1-3* nodules, which probably correlates with the lower number of infected cells, and the reduced zonation of *dnf1* mutant nodules possessing mainly an enlarged invasion zone [7]. This result is in agreement with the observation that *NAD1* expression was reduced in wild-type plants nodulated with *bacA* mutant rhizobia. Taken together, these results indicate that the defense-like responses were induced irrespectively of the differential phase of rhizobia in *nad1-3* nodules, similarly to what was found in the *dnf2* mutant [23].

## 4. Conclusions

The establishment of successful root nodule symbiosis requires the suppression of plant immunity in multiple steps during the symbiotic interaction with nitrogen fixing microbes [18,61,62]. Once rhizobia are released from infection threads, bacteria colonize nodule cells remaining separated from the plant cytoplasm by the plant-derived symbiosome membrane. Following multiplication and differentiation, infected nodule cells accommodate thousands of bacteria, and, therefore, the regulation of plant defense responses in nodules is critical for the colonization of host cells. Several genes that are involved in the control of defense reactions in symbiotic nodules upon the internalization of rhizobia have been recently identified [19,20,21,22]. Nodules of mutant plant defective in *NAD1*, display uncontrolled plant defense responses, such as accumulation of phenolic compounds, increased level of hydrogen peroxide, and the activation of defense-related genes ,which finally resulted in the necrosis of rhizobial and host cells [21].

Here, we described and functionally analyzed two novel mutant alleles of the *NAD1* gene. Both the *nad1-3* deletion and *nad1-4* insertion mutants showed the very similar ineffective symbiotic phenotype and displayed brownish pigmentation in nodules corresponding to the accumulation of phenolic compounds, indicating the induction of defense-associated response, as reported previously in *nad1* mutants [21].

The results presented here help us to better understand the functioning of NAD1 in the suppression of plant defense responses during the rhizobial nitrogen-fixing symbiotic interaction. The epistasis analysis using ineffective symbiotic mutants of *M. truncatula*, nodulated with rhizobia defective to form functional nitrogen fixation, suggests the hierarchy of plant mutants in the symbiotic process. Based on these results, the suppression of plant defense responses that are controlled by NAD1 occurs irrespectively of the differentiation status of bacteroids at the early stage of intracellular colonisation of rhizobia. We also presented that NAD1 functions after the release of rhizobia from infection threads, and that activity of NAD1 is required in the infected cells of the functioning nodules.

## Supplementary Materials

The following are available online at www.mdpi.com/2073-4425/8/12/387/s1. Figure S1: The symbiotic phenotype of *nad1-3* and wild-type (Jemalong) plants, Figure S2: Positional cloning of the *nad1-3* locus and confirming the mutations in *nad1* alleles, Figure S3: Complementation experiments of *nad1-3*, Figure S4: Time course analysis of accumulation of phenolic compounds in *nad1-3*, *dnf2-1* and *nad1-3/dnf2-1* mutants, Figure S5: Viability staining of *S. medicae* WSM419 rhizobia in *nad1-3*, *dnf2-1* and *nad1-3/dnf2-1* mutant nodules, Figure S6: Ultrastructure of the wild-type and *nad1-3* nodules, Table S1: *M. truncatula* lines and rhizobial strains used in this study, Table S2: Primers used in this study [37,38].
