# Supplementary material for: NAD1 Controls Defense-Like Responses in Medicago truncatula Symbiotic Nitrogen Fixing Nodules Following Rhizobial Colonization in a BacA-Independent Manner
Source: Genes (Basel). 2017 Dec 14;8(12):387. doi: 10.3390/genes8120387 (PMC5748705; doi:10.3390/genes8120387)
Supplement: Supplementary file 1 [file genes-08-00387-s001.pdf]

NAD1 controls defense-like responses in *Medicago truncatula* symbiotic nitrogen fixing nodules following rhizobial colonization in a BacA-independent manner.

Ágota Domonkos[1], Szilárd Kovács[2,3], Anikó Gombár[1], Ernő Kiss[3], Beatrix Horváth[1], Gyöngyi Z. Kováts[1], Attila Farkas[2], Mónika T. Tóth[1], Ferhan Ayaydin[4], Károly Bóka[5], Lili Fodor[1], Pascal Ratet[6,7], Attila Kereszt[2], Gabriella Endre[2,3] and Péter Kaló[1]

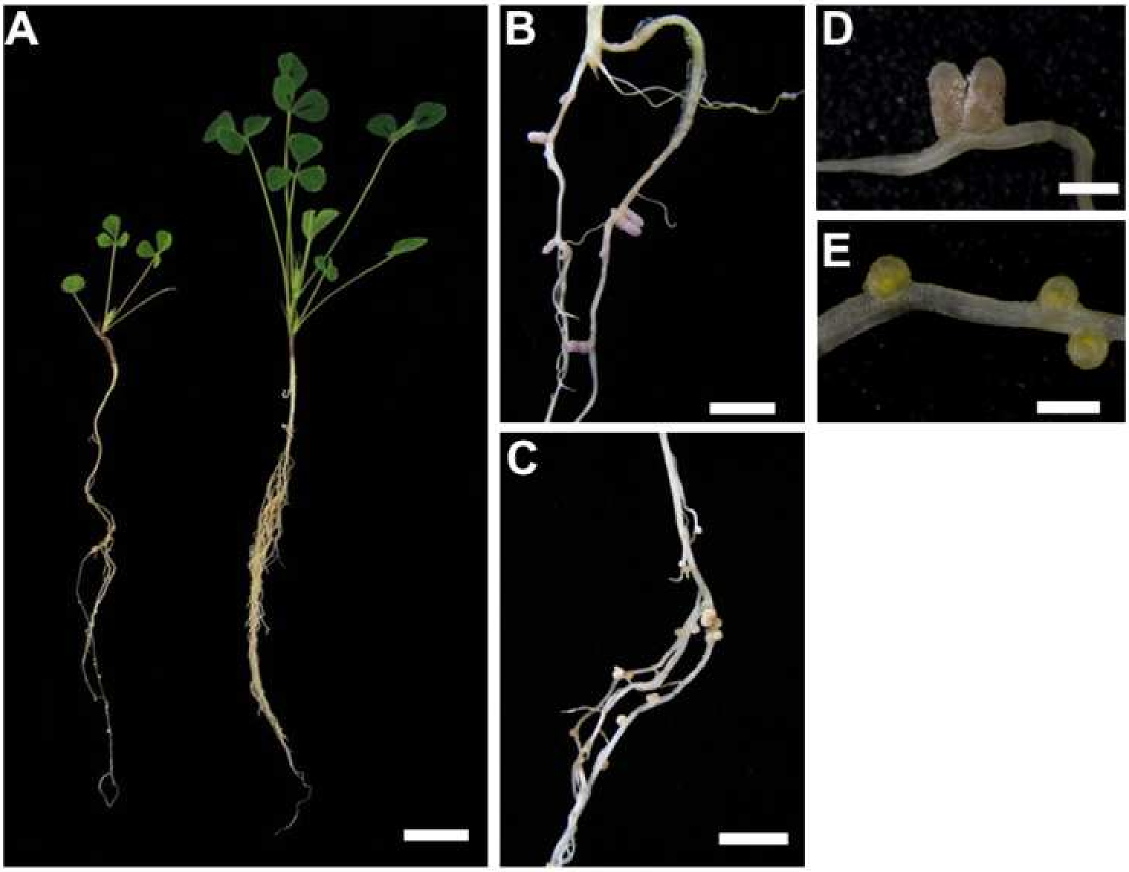

Figure S1. The symbiotic phenotype of *nad1-3* and wild-type (Jemalong) plants 18 dpi with *S. medicae* strain WSM 419. Ineffective symbiotic mutant displayed the symptoms of nitrogen starvation (reduced growth) when grown under symbiotic conditions (A). Pink elongated nodules characteristic of an efficient symbiosis were observed on wild-type roots (B and D). *nad1-3* mutant developed small slightly spherical and brownish nodules (C and E). Scale bars: A 20 mm, B and C 5 mm, D and E 1 mm

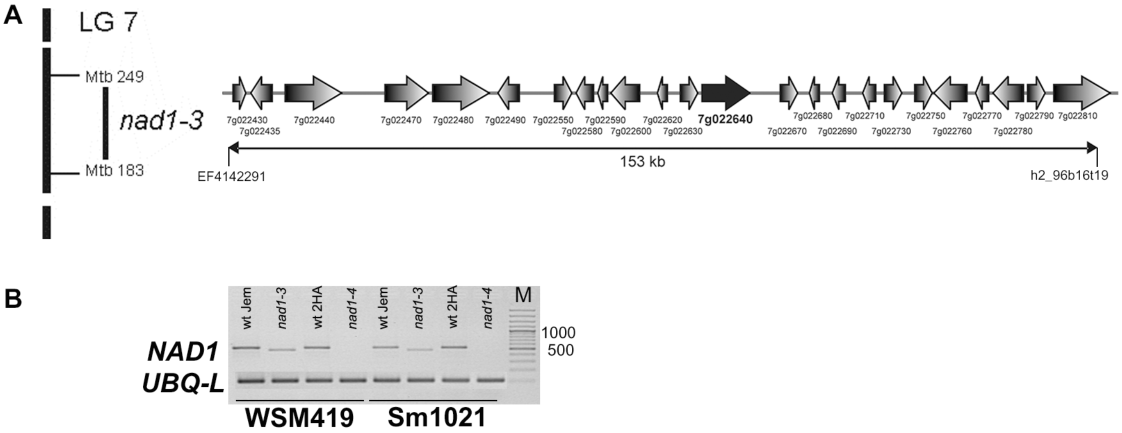

Figure S2. Positional cloning identified the mutant locus of *nad1-3* between the genetic markers MtB249 and MtB183 on chromosome 7 (A). Fine mapping defined a genomic region of 153 kb containing 24 gene models represented by arrows, including the *NAD1* (*7g022640,* black arrow) gene, based on the genome assembly of *M. truncatula.*

RT-PCR products of *nad1* and wild-type (Jemalong accession and 2HA genotype) nodules induced either by *S. medicae* WSM419 (WSM419) or *S. meliloti* strain 1021 (Sm1021) indicate the absence of *NAD1* expression in *nad1-4* (B). RT-PCR products were generated using a primer pair located on the first and the second exon of *NAD1* from nodule samples 14 dpi. The size of the PCR fragment is the same in the wild-type Jemalong and 2HA parents of the *nad1-3* and *nad1-4* mutant lines, respectively. We could not detect *NAD1* transcript in *nad1-4* nodules and the RT-PCR produced a smaller fragment from *nad1-3* compared to the wild-type due to the 50-bp deletion.

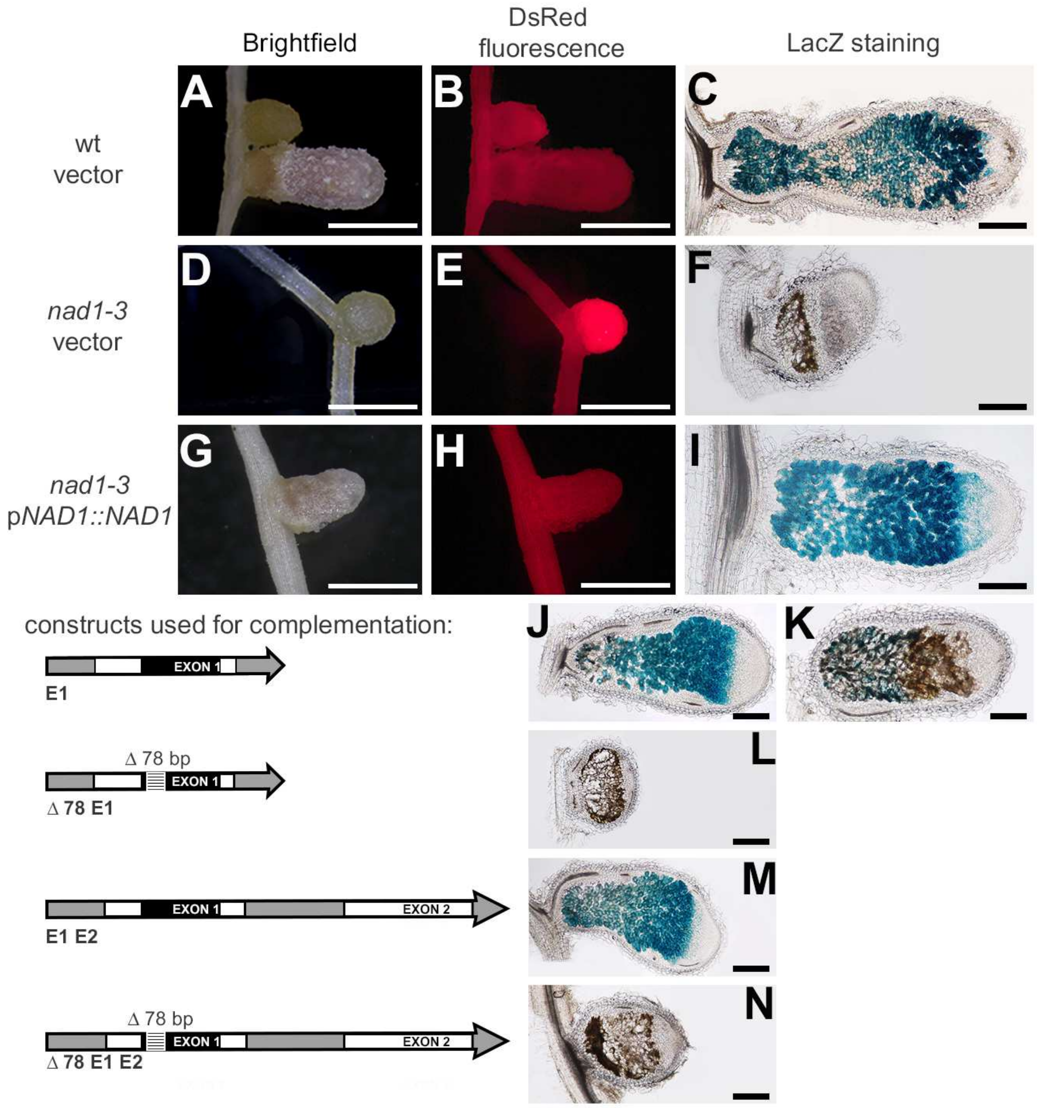

Figure S3. (A-N) Both coding and non-coding exons of *NAD1* are required for efficient restoration of the nodulation phenotype of *nad1-3*. The *NAD1* gene was introduced into *nad1-3* with *Agrobacterium rhizogenes* and transformed roots were inoculated with *S. medicae* WSM419 (pXLGD4). Complementation was assessed by the disappearance of the brown pigmentation typical for *nad1* mutants and colonization of the zones of the indeterminate nodules. Transgenic roots were identified by red fluorescent protein. Nodules on transgenic roots of wild-type (A-C) and *nad1-3* (D-F) plants induced by the empty vector showed the nodule phenotype typical for wild-type and *nad1-3* mutant plants, respectively. Mixture of nodules presenting the absence or the presence of brown pigmentation were developed on roots transformed with the construct containing the first exon of *NAD1* indicating the partial complementation of *nad1-3* (J and K). Complementation of the *nad1-3* mutant was carried out with the construct containing both exons of *NAD1* (G-I and M). The constructs containing an intron predicted based on the BG582085 EST sequence failed to restore the nodulation phenotype of *nad1-3* (L and N). Both wild type and nodules with brown pigmentation were developed on roots transformed with the construct containing the first exon of *NAD1* indicating the partial complementation of *nad1-3*. In all construct the *NAD1* gene and its deletion versions were driven by *NAD1* native promoter. Arrows indicate the composition of the construct used for transformation. Grey bars show promoter and intronic regions, grey arrows present the 3' UTR, blank bars show exons and black regions display the coding sequence of *NAD1*. Striped boxes show the intronic sequence presumed based on the EST BG582085. Scale bars: A, B, D, E, G and H 1 mm, C, F, I, J-N 200 μm

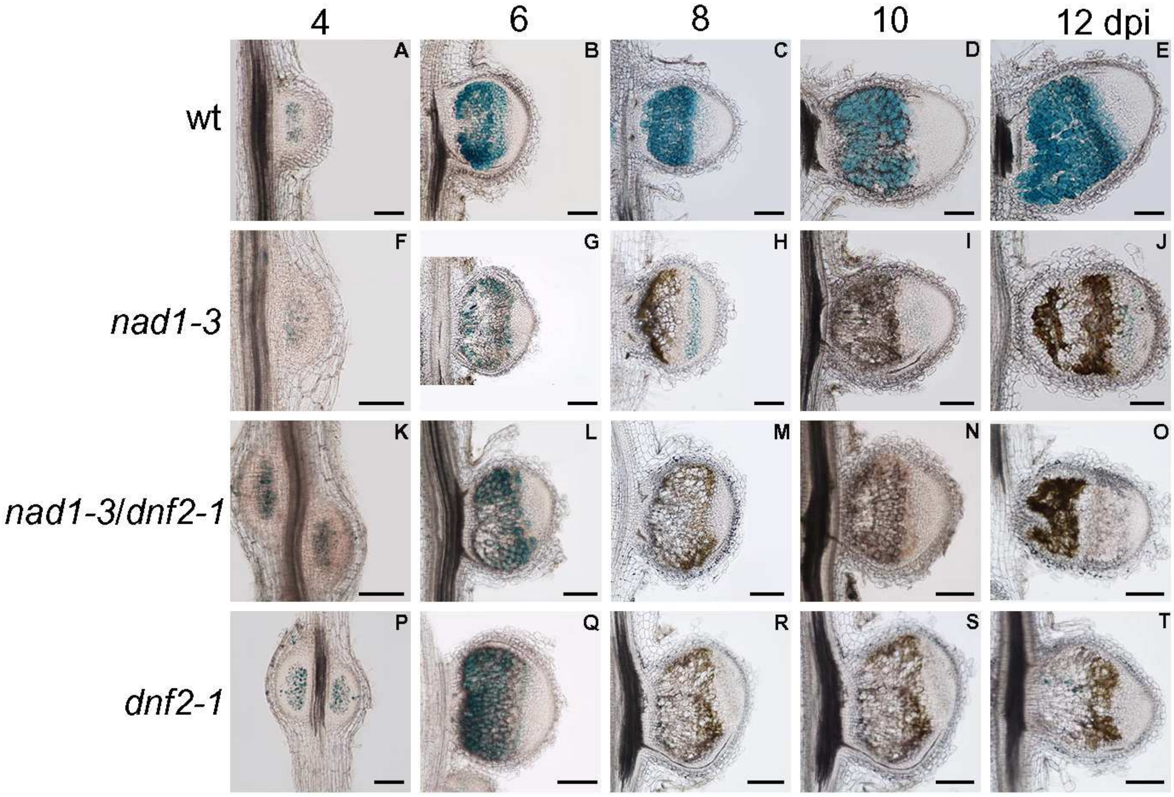

Figure S4. The appearance of brown pigmentation is synchronized in *nad1-3*, *dnf2-1* and *nad1-3/dnf2-1* mutants. Nodule sections were stained for β-galactosidase activity after 4, 6, 8, 10, and 12 dpi with *S. medicae* WSM419 (pXLGD4). The production of brown pigments (natural color), detected with no staining for pigmentation in these sections, corresponding to phenolic compounds (Figure 4) is induced between 6 and 8 dpi only in the mutant nodules. Note that the blue β-galactosidase staining observed at 6 dpi is only maintained in wild-type nodules at 8, 10 and 12 dpi. wt (wild-type, panel A-E), *nad1-3* (panel F-J), *nad1-3/dnf2* (panel K-O) and *dnf2* (panel P-T). Nodules were harvested at 4 (A-P), 6 (B-Q), 8 (C-R), 10 (D-S) and 12 dpi (E-T). Scale bars: 200 µm

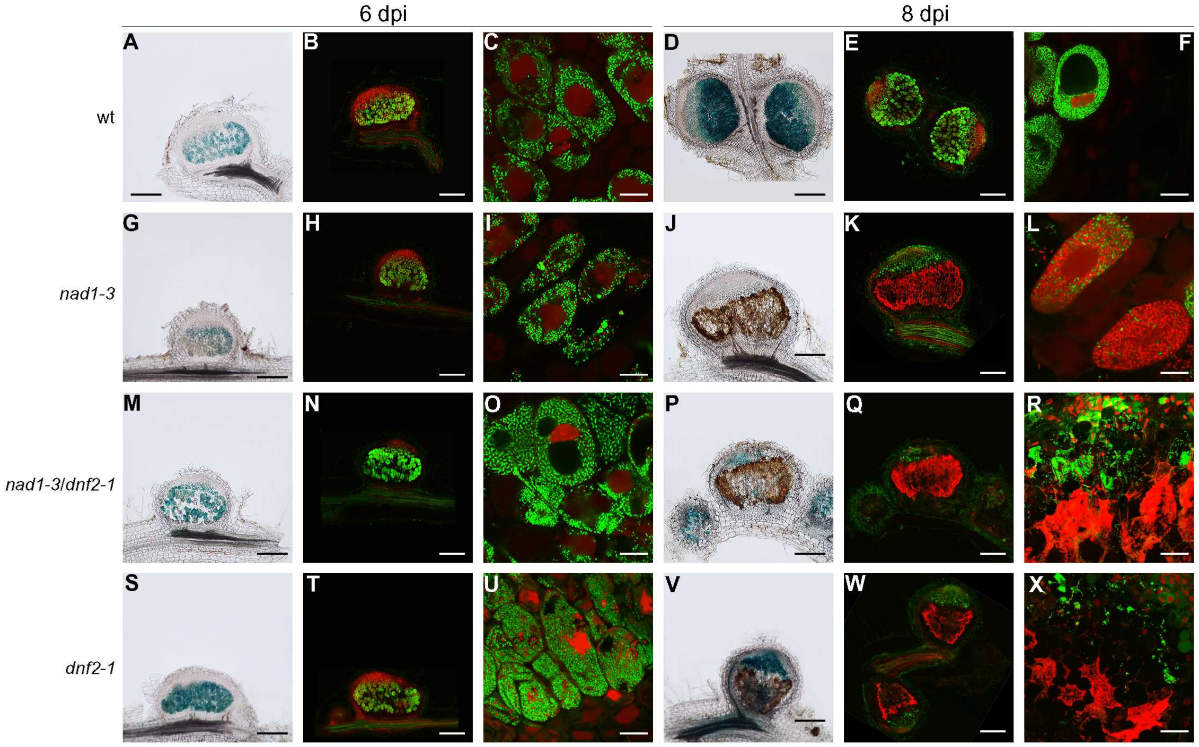

Figure S5. Live/dead staining of *S. medicae* WSM419 rhizobia indicates rapid death of bacteria in *nad1-3*, *dnf2-1* and *nad1-3/dnf2-1* mutant nodules. Mutant and wild-type nodules stained for β-galactosidase activity (A, D, G, J, M, P, S and V) and with a mixture of SYTO9 (green signal) and propidium iodide (red signal in plant nuclei) (B, C, E, F, H, I, K, L, N, O, Q, R, T, U, W and X) were indistinguishable 6 dpi (columns 1 to 3) but mutant phenotype appeared 8 dpi (columns 4-6). Strong autofluorescence, pseudocolored in red, was observed in the proximal part of mutant nodules and few cells with dead bacteria fluorescing red were found in the transition zone between the colonized host cells and the area showing autofluorescence indicating the rapid necrosis of rhizobia in mutant nodules (L, R, X).

Wild-type nodules: A-F, *nad1-3* nodules: G-L, *dnf2* nodules: M-R, *nad1-3/dnf2* nodules: S-X

Scale bars: A, B, D, E, G, H, J, K, M, N, P, Q, S, T, V and W 200 μm, C, F, I, L, O, R, U and X 20 μm

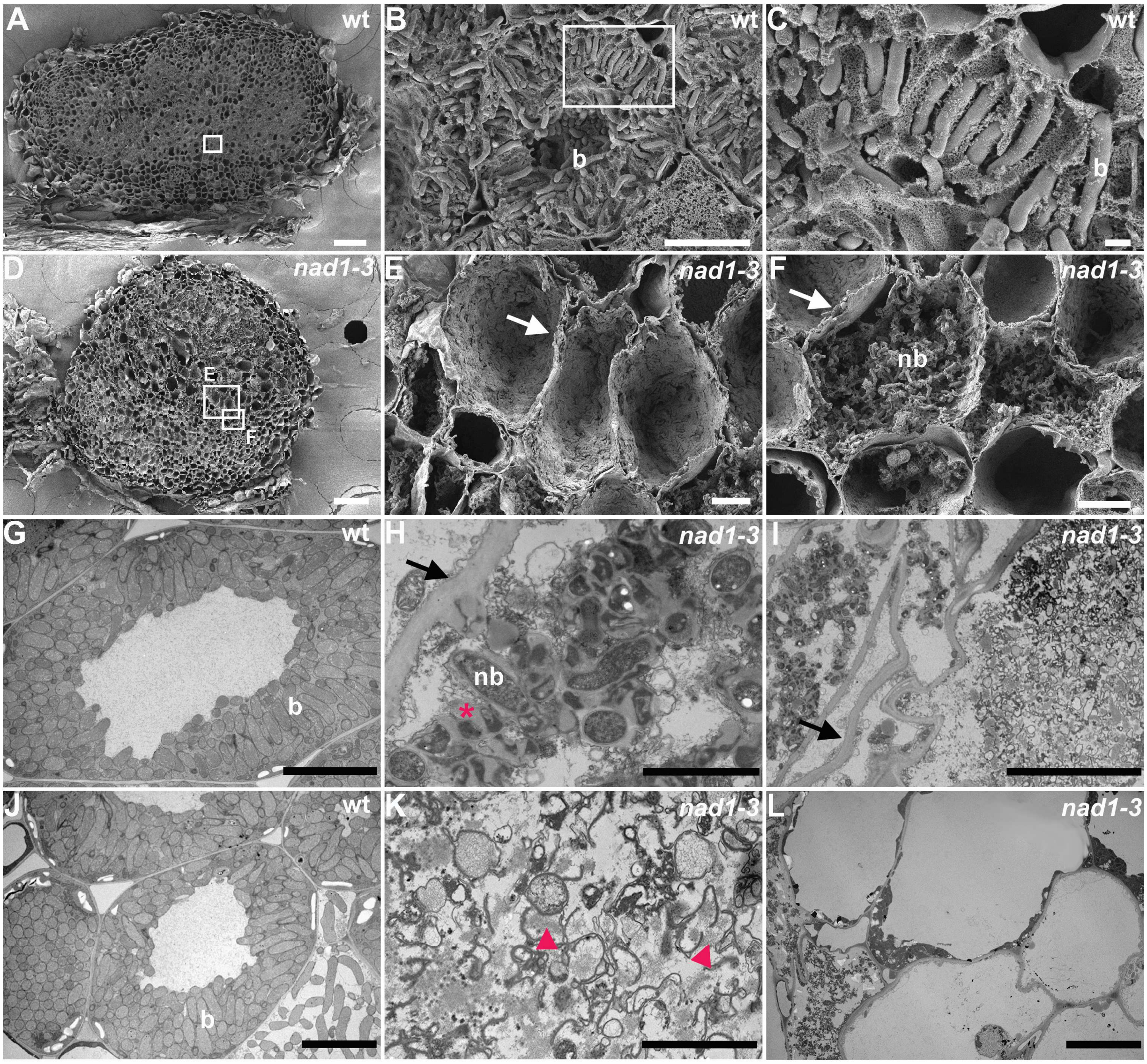

Figure S6. Symbiotic cells in *nad1-3* nodules undergo necrosis. Scanning electron microscope images of 8-dpi wild type nodules show the symbiotic cells colonized by elongated bacteroids (A-C). In *nad1-3* nodules cells devoid of bacteria or containing necrotic bacteria were detected (D-F). The transmission electron microscope images of wild-type nodule at 18 dpi show differentiated bacteria orientated towards the central vacuole in a cell of the nitrogen fixation zone (G and J). Nodule cells in *nad1-3* nodules undergo rapid decomposition (H, I, K and L) resulting in empty cells (L). Boxes on panels A, B and D show magnified regions presented in panels B, C and E, F, respectively. b: differentiated bacteroids (B, C G and J) nb: necrotic bacteria (F and H) arrows: thickened cell walls (E, F, H and I); asterisk: thickened plant cell walls surrounding necrotic bacteria (H); arrowheads: hydrolyzed cell wall remnants (K). Scale bars: A and D 100 µm, B, E, F, G, I, J and L 10 µm, C 1 µm, H and K 2 µm

A

Medicago truncatula **line**	**Features**	**Reference**
Jemalong	wild-type control in analyses of *nad1-3*	
A20	wild-type crossing partner in genetic mapping	
2HA	wild-type control in analyses of *nad1-4*	
nad1-3	Jemalong background	[35]
nad1-4	Jemalong genotype 2HA background	this study
dnf2-1	Jemalong background	[19, 45]
ipd3-1	Jemalong background	[60]
dnf1-1	Jemalong background	[7]
lin-2	Jemalong genotype A17 background	[59]

B

**Rhizobial strain**	**Features**		**kindly provided by**
Sinorhizobium (Ensifer) meliloti 1021	wild-type		
Sinorhizobium (Ensifer) medicae WSM419 (pXLGD4)	wt bacteria constitutively expressing *lacZ* reporter gene		J. Terpolilli (Murdoch University).
Sinorhizobium (Ensifer) medicae WSM419 (pMEpTrpGFPGUS)	wt bacteria constitutively expressing the β-glucuronidase gene	this study	
Sinorhizobium (Ensifer) meliloti 1021 *nodA* mutant	deficient in nodulation factor (NF) production	this study	
Sinorhizobium (Ensifer) meliloti 1021 *exoY* mutant	mutant in succinoglycan (EPS-I) production	[16]	Hai-Ping Cheng (Univ. New York)
Sinorhizobium (Ensifer) meliloti 1021 *bacA mutant* (Δ*bacA* null mutant; *bacA654::Spc*)	transport of macromolecules; deficiency in bacteroid development	[29]	Graham C. Walker (MIT)

Table S1 A and **B**. *M. truncatula* lines and rhizobial strains used in this study

	Forward primer 5'-3'	Reverse primer 5'-3'	Reference	Gene ID
**primers used for q RT-PCR**				
7Y - NAD1	GTGTTGTGGGCAGGATTTGGC	CCATCCAAAGGTGCAGGTGC	this study	*Medtr7g022640*
UBP (Ubiquitin-like protein)	GGCCCTAGAACATTTCCTGTGG	CAGTCTTCAAAACTCTTGGGCAG	[37]	*Medtr3g091400*
PTB	CGCCTTGTCAGCATTGATGTC	TGAACCAGTGCCTGGAATCCT	[37]	*Medtr3g090960*
chitinase	GGGCTTGAATGCGGAAGAGG	CAAGATTGTCTCCATATCCAACTCC	[38]	*Medtr3g118390*
NDR1	GGGAAATTGAAGCTTCCCAAAAT	CCTAAACCTAAATTTACAACTACTGCTCC	[38]	*Medtr5g076170*
flavonol synthase	CACCGATGCTTTTGTCATAAACG	TGAATAATGCAGGCCTTTCAGG	this study	*Medtr5g055680*
PR10	TGTTGAAGATGGTGAGACCAAGC	GTCTGGAAGGCCAACACCTCC	this study	*Medtr2g035150*
plant invertase	TTTGGGTAAGGCTAAGGGAGAGG	TGCTTGGAGGCAAAGTTATTAACC	this study	*Medtr4g101760*
Kunitz type trypsin inhibitor	TATCATACTACGGTGTGGAAGC	CTGGTAGGGAACATCACTAAGAG	this study	*Medtr6g078250*
**primers used for confirmation of *M. truncatula* double mutants**				
lin-2 mut F-R	ACATATGAGTTCAATTGTAGGGTGG	GGAACAACCTTCTTGCTTGAGG	this study	*Medtr1g090320*
7Y F8-R8	TATTCTCATCTTGTTTACACCTTTGG	GCCAAATCCTGCCCACAACAC	this study	*Medtr7g022640*
IPD3 F13-R13	ATCCTGTTGATAGAGA	GAGTATGAAGATTTATTG	[60]	*Medtr5g026850*
DNF1 F1-R1	CAATGAGACCCCACCGAATAC	GACACATCAACACCGACAATAAT	this study	*Medtr3g027890*
**primers used for cloning and expression studies**				
7Y_GWF6	ggggacaagtttgtacaaaaaagcaggctGGCTCAATCCATCATAGTGTAT		this study	*Medtr7g022640*
7Y_GWFR6	ggggaccactttgtacaagaaagctgggtCACTGCACTTGTTATTGTAGCACC		this study	*Medtr7g022640*
7Y_promGWR	ggggaccactttgtacaagaaagctgggtCTTTTTTTTCTTCTTTTCTCAAGATACAATTATG		this study	*Medtr7g022640*
7Y_GWR10	ggggaccactttgtacaagaaagctgggtAAAGAGTTATAAGGAAACTCTCAAAATAAG		this study	*Medtr7g022640*
7Y_F9	TCCATTTCGTAAAGCAAATTTCAG		this study	*Medtr7g022640*
7Y_R9	CATTTAACATCTTTTTTTTCTTCTTTTCTC		this study	*Medtr7g022640*
7Y_F8	TATTCTCATCTTGTTTACACCTTTGG		this study	*Medtr7g022640*
7Y_qR6	GGGACATCATAGTATGGGTCTTGC		this study	*Medtr7g022640*
*Medtr7g022640*_Fstart	ATGTTAAATGGTAAAGAAAAAAATTGC		this study	*Medtr7g022640*
*Medtr7g022640*_Rend	ATCTCCATCCAAAGGTGCAGGTG		this study	*Medtr7g022640*
*Medtr7g022640.1*_F5UTR	GTTAGTGCCAAGATCACATATATTC		this study	*Medtr7g022640*
*Medtr7g022640.1*_Rstop	CTAATCTCCATCCAAAGGTGCAG		this study	*Medtr7g022640*

Table S2. Primers used in this study. Small characters in primer sequences indicate nucleotides of the attenuation sites required for Gateway cloning.
